# Clarifying Methodological Principles and Preliminary Trends in Digital Vestibular Rehabilitation: Authors’ Reply

**DOI:** 10.2196/109396

**Published:** 2026-09-30

**Authors:** Azriel Kaplan, Liran Kalderon, Amit Wolfovitz, Yoav Gimmon, Shelly Levy-Tzedek

**Affiliations:** 1 Department of Physical Therapy, Recanati School for Community Health Professions, Ben-Gurion University of the Negev, David Ben-Gurion Blvd 1, Beer-Sheva, Southern District, 8410501, Israel, 972 86479563; 2 Department of Otolaryngology—Head and Neck Surgery, Sheba Medical Center, Ramat Gan, Tel Aviv, Israel; 3 Department of Physical Therapy, Faculty of Social Welfare & Health Sciences, University of Haifa, Haifa, Haifa, Israel; 4 Zelman Center for Neuroscience, Ben-Gurion University of the Negev, Beer-Sheva, Southern District, Israel; 5 Freiburg Institute for Advanced Studies (FRIAS), University of Freiburg, Freiburg, Baden-Wurttemberg, Germany

**Keywords:** vestibular rehabilitation, smartphone, crossover trial, digital health, statistical analysis

## To the Editor

We welcome the commentary [1] regarding our publication [2] evaluating the Vestibulon app in vestibular rehabilitation. We appreciate the opportunity to clarify the clinical and statistical considerations guiding our pilot trial and to contextualize our findings.

The author raises a concern regarding the absence of a washout period. The absence of a washout period was a deliberate clinical decision. Vestibular rehabilitation involves central compensation, adaptation, habituation, and motor learning, which may not be reliably reversed by a short interruption. Because participants were symptomatic and receiving clinically indicated care, continuity of rehabilitation was prioritized. We nevertheless acknowledge that carryover from the first period may have limited the interpretation of period-2 comparisons, as noted in our Limitations section [2].

Given the pilot nature of the study and the small sample size, we used prespecified nonparametric methods for the exploratory comparisons. This approach was appropriate for the bounded and potentially skewed Dizziness Handicap Inventory (DHI) and State-Trait Anxiety Inventory scores, which derive from ordinal response categories, and was applied consistently to the continuous Instrumented Timed Up and Go (iTUG) measure. We acknowledge that these tests do not constitute a complete crossover analysis accounting for treatment, period, sequence, within-participant correlation, and potential carryover. This exploratory analysis was not intended to provide definitive evidence of the app’s efficacy, but rather to provide initial clinical insights and establish feasibility for future studies.

Across all sections, we explicitly refrained from making definitive claims of superiority, including regarding early iTUG changes, repeatedly framing results as “preliminary trends.” Importantly, our Limitations section explicitly outlined the constraints noted by the author: the pilot sample size, potential carryover effects due to the absence of a washout period, and the essential need for a future fully powered randomized controlled trial (RCT).

We thank the author for identifying the typographical error on page 9, where *P*=.057 was inadvertently reported twice. This *P* value (.057) corresponds to the 2-sided Fisher exact test for the week 6 comparison of 7 of 9 versus 2 of 8 minimal clinically important difference (MCID) responders. Furthermore, for period-specific responder outcomes (6/17 versus 3/17), a valid paired analysis relied on discordant pairs, with 5 participants achieving the MCID strictly during the app period and 2 strictly during the conventional period (exact McNemar test: *P*=.453). Therefore, they should not be analyzed as independent samples alone. As a supplementary analysis less affected by carryover, we analyzed period 1 using an analysis of covariance. Baseline-adjusted week 1 DHI scores were 38.82 in the app-first group and 40.33 in the conventional-first group, a difference that was not statistically significant (*F*=0.04, *P*=.840, partial η^2^=.002). The DHI MCID was achieved by 5 of 9 versus 1 of 8 eligible participants (55.6% vs 12.5%; *P*=.131). Although exploratory for definitive inference, these findings provide a useful trend and preliminary data for an RCT.

In conclusion, our pilot trial supports the feasibility of integrating smartphone support into vestibular rehabilitation and observed improvement in functional outcomes over the 6-week rehabilitation program. We agree that establishing definitive incremental efficacy requires a parallel-group RCT, and we thank the author for this constructive scientific dialogue.

## Abbreviations

DHI: Dizziness Handicap Inventory
iTUG: Instrumented Timed Up and Go
MCID: minimal clinically important difference
RCT: randomized controlled trial
